# Culture, Age, and Self-Continuity: Old Chinese Showed Lower Continuity With Their Past and Future Self Than Americans

**DOI:** 10.1093/geroni/igaa057.1450

**Published:** 2020-12-16

**Authors:** Yi Lu, Lu Cong, Corinna Loeckenhoff, Xin Zhang

**Affiliations:** 1 Peking University, Beijing, China; 2 Cornell University, Ithaca, New York, United States

## Abstract

As culture shapes the way people think and reason, it may also influence their perception of self-continuity, the psychological proximity to the past and the future, across the lifespan. Meanwhile, previous studies in America indicated that advancing age was associated with greater self-continuity. The present research is the first to simultaneously examine how age and culture interact with each other on individuals’ continuity with past and future self. Using Ersner-Hershfield’s visual scale, we assessed participants’ temporal self-continuity at 3 past and 3 future time points (1 year vs. 5 years vs. 10 years) in a sample of 375 Chinese and 91 Americans. A 2(age: young vs. old) x 2(temporal direction: past vs. future) x 2(culture: Chinese vs. American) multilevel analysis was conducted. A significant interaction of age and culture was found, and such interaction revealed that younger Chinese and Americans shared a similar pattern on self-continuity at different temporal distances. However, older Chinese, compared with older Americans, presented a lower level of self-continuity and less variance across temporal distances, suggesting that older Chinese felt less connected with their recent self than both Americans and younger Chinese, and less connected with their remote self than older Americans. These findings fill the gaps in current research by revealing an opposite trend on self-continuity between older Chinese and Americans, and suggest more concern on country differences in this area.

